# Ageing of enteric neurons: oxidative stress, neurotrophic factors and antioxidant enzymes

**DOI:** 10.1186/1752-153X-6-80

**Published:** 2012-08-02

**Authors:** Kris Korsak, Nazanin F Dolatshad, Ayona T Silva, M Jill Saffrey

**Affiliations:** 1 Department of Life, Health and Chemical Sciences, Biomedical Research Network, The Open University, Milton Keynes MK7 6AA, UK; 2 The RoyalVeterinaryCollege, Hawkshead Lane, Hatfield, Hertfordshire, AL9 7TA, UK

**Keywords:** Myenteric plexus, Ileum, Intestine, Rat, Caloric restriction, NT-3, GDNF, Superoxide dismutase, Catalase

## Abstract

**Background:**

Ageing is associated with gastrointestinal dysfunction, which can have a major impact on quality of life of the elderly. A number of changes in the innervation of the gut during ageing have been reported, including neuronal loss and degenerative changes. Evidence indicates that reactive oxygen species (ROS) are elevated in ageing enteric neurons, but that neurotrophic factors may reduce generation of neuronal ROS. Two such factors, glial cell line derived neurotrophic factor (GDNF) and neurotrophin-3 (NT-3) have also been found to protect enteric neurons against oxidative stress induced cell death of enteric ganglion cells *in vitro.* We have investigated the possible roles of neurotrophic factors further, by examining their expression in the gut during ageing, and by analysing their effects on antioxidant enzyme production in cultures of enteric ganglion cells.

**Results:**

Analysis of the expression of GDNF and its receptors c-Ret and GFR α − 1 in rat gut by RT-PCR showed that expression continues throughout life and into ageing, in both *ad libitum*(AL) and calorically-restricted (CR) animals. Levels of expression of GDNF and GFR α − 1 were elevated in 24 month AL animals compared to 24 month CR animals, and to 24 CR and 6 month control animals respectively. The related factor Neurturin and its receptor GFR α − 2 were also expressed throughout life, the levels of the GFR – α-2(b) isoform were reduced in 24 m AL animals. Immunolabelling showed that c-Ret and GFR α − 1 proteins were expressed by myenteric neurons in ageing animals. GDNF, but not NT-3, was found to increase expression of Cu/Zn superoxide dismutase and catalase by cultured enteric ganglion cells.

**Conclusions:**

The neurotrophic factors GDNF and neurturin and their receptors continue to be expressed in the ageing gut. Changes in the levels of expression of GDNF , GFR α-1 and GFR α-2(b) isoform occurred in 24 m AL animals. GDNF, but not NT-3, increased the levels of antioxidant enzymes in cultured enteric ganglion cells, indicating a possible mechanism for the reported protective effect of GDNF against menadione-induced neuronal apoptosis in the ageing gut. Together these data suggest that GDNF family members may play a protective role in the gut throughout life, and support the suggestion that dysregulation of neurotrophic factor support could contribute to neuronal ageing in the gut.

## Background

The gastrointestinal (GI) tract is a complex organ system. It is composed of many different cell types, the co-ordinated functions of which are essential for normal GI function. Ageing is associated with increased incidence of several GI disorders, including constipation and incontinence, and, in the small intestine, impaired absorption of nutrients, all of which have major impact on quality of life and healthcare costs [1,2]. Age-associated changes have been described in several different intestinal cell types, including intestinal epithelial stem cells [3], smooth muscle [1] and the intrinsic and extrinsic neurons that innervate the gut and regulate GI functions. With regard to nervous system changes, neurodegeneration of both intrinsic and extrinsic neurons has been reported see [2,4-6].

Analysis of changes in the intrinsic enteric ganglia during ageing has shown that caloric restriction may protect against neurodegeneration [7,8]. Investigation of the possible mechanisms underlying this protective effect showed that generation of reactive oxygen species (ROS) was reduced by neurotrophin-3 (NT-3) and glial cell line-derived (GDNF) treatment in enteric neurons from calorically restricted (CR) animals [8]. In addition, these factors were found to protect enteric neurons from oxidative stress; apoptosis in response to menadione treatment being reduced in the presence of both NT-3 and GDNF [8]. Cell culture models of the enteric nervous system (ENS) have also been used to analyse these protective effects; NT-3 has been found to protect enteric ganglion cells from hydrogen peroxide-induced toxicity [9].

In the present study we have investigated the protective effects of these factors further, addressing two main questions. First, to determine if the protective factor GDNF and related factor Neurturin (NTN), their signalling receptor c-RET and binding receptors GFRα − 1 and GFR α − 2 continue to be expressed in the ageing gut of *ad libitum*(AL) and CR rats, and second, to investigate the possible mechanism underlying their protective actions, by examining if GDNF, and also NT-3, have an effect on the levels of antioxidant enyzmes in cultured enteric ganglion cells.

## Results and discussion

### Expression of GDNF, Neurturin and their receptors in the ageing gut

**Figure 1:**
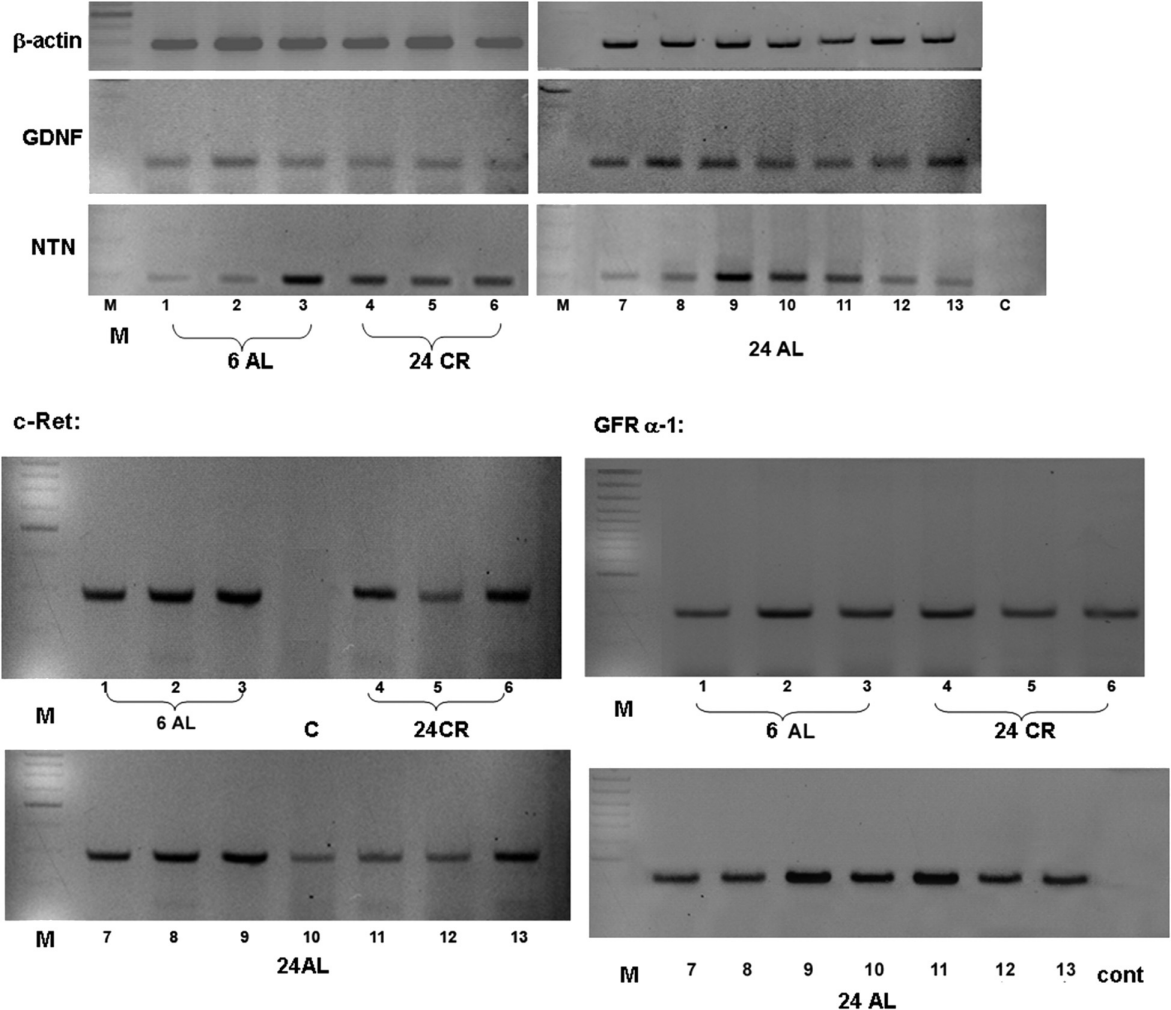
**Expression of GDNF and NTN mRNA and that of the receptors c-Ret and GFRα-1 in the muscularis externa of 6 month AL-fed rats (6AL, n = 3) and 24 month CR rats (24CR, n = 3) and 24 AL-fed rats (24AL, n = 7).** Expression of β-actin was used for external standardisation. A negative control (C) was run, in which the RT enzyme was omitted. M = molecular markers.

**Figure 2:**
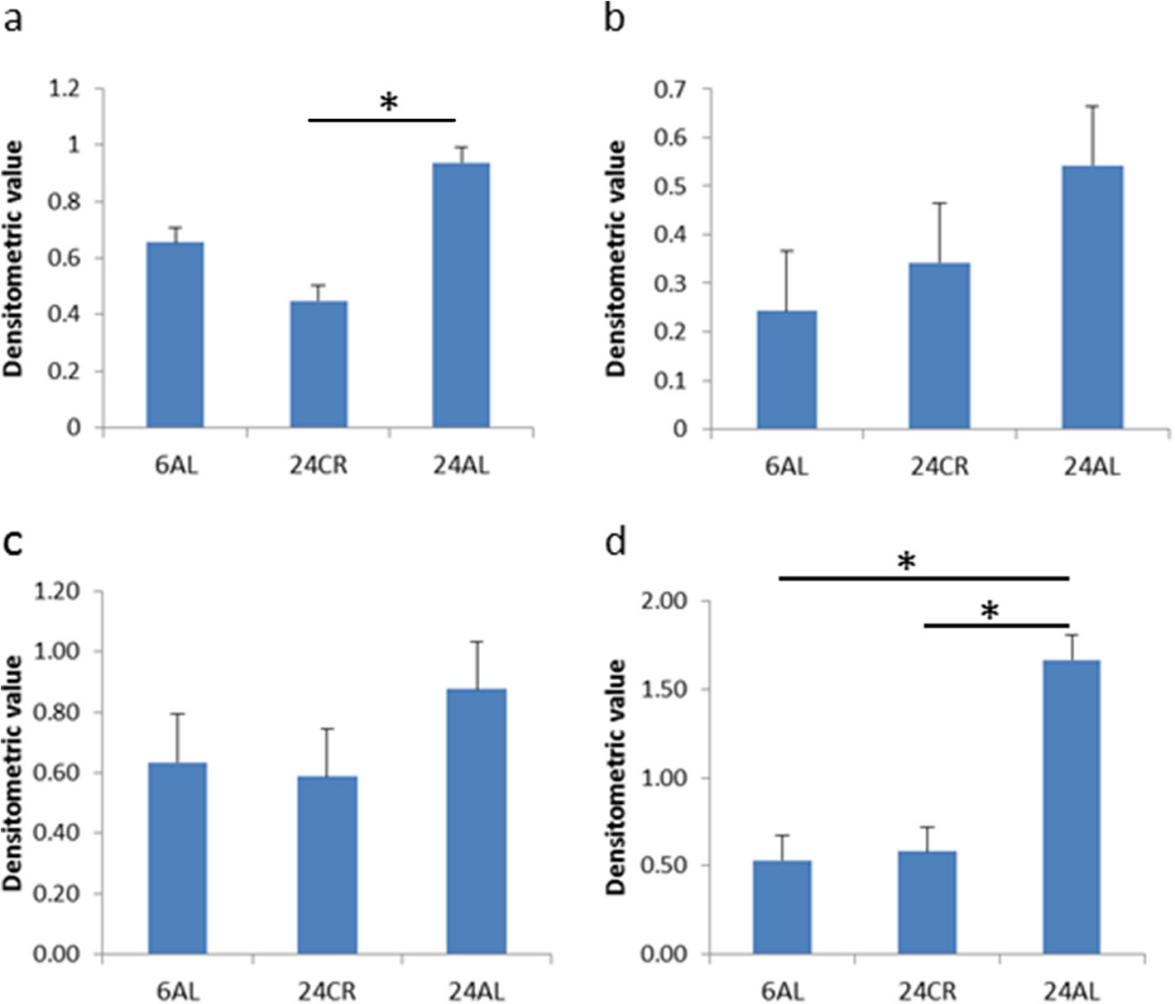
**Densitometric analysis of (a) GDNF (b) NTN (c) c-ret (c) and (d) GFR α-1 RT-PCR gels standardised against β-actin expression.** Levels are in arbitrary units, bars show standard errors. * p ≤ 0.05.

The expression of GDNF, Neurturin and their receptors were analysed by methods described in Additional file 1. GDNF and the related factor NTN mRNAs were found to be expressed in the muscularis externa of animals of both 6 months and 24 months of age (Figure 1). Densitometric analysis showed that GDNF levels were significantly elevated in 24 month AL-fed animals compared to 24 month CR-fed animals (Figure 2a). No differences in NTN expression in samples from AL and CR 24 month old animals were observed (Figure 2b), although there appeared to be more variation in NTN transcript levels in samples from AL-fed animals (Figure 1).

GDNF and NTN act via a receptor complex involving two receptors; a signalling receptor, c-Ret (used by all members of the GDNF family), and a glycoyslphosphatidyl inositol (GPI)-linked binding receptor; GFR α-1 for GDNF and GFR α-2 for NTN. The expression of mRNA encoding these receptors was also studied. The primers used to analyse GFR α-2 expression were chosen to allow all three splice isoforms to be detected [10]. Transcripts for all three receptors were detected in the muscularis externa of all adult and aged rats. c-Ret expression was maintained in ageing animals fed either a CR or AL diet (Figure 1), and no difference in expression levels in the different groups was measured (Figure 2c). There appeared to be some variation in the level of expression of c-Ret transcripts in both 24 month CR and 24 month AL-fed animals (Figure 1).

**Figure 3:**
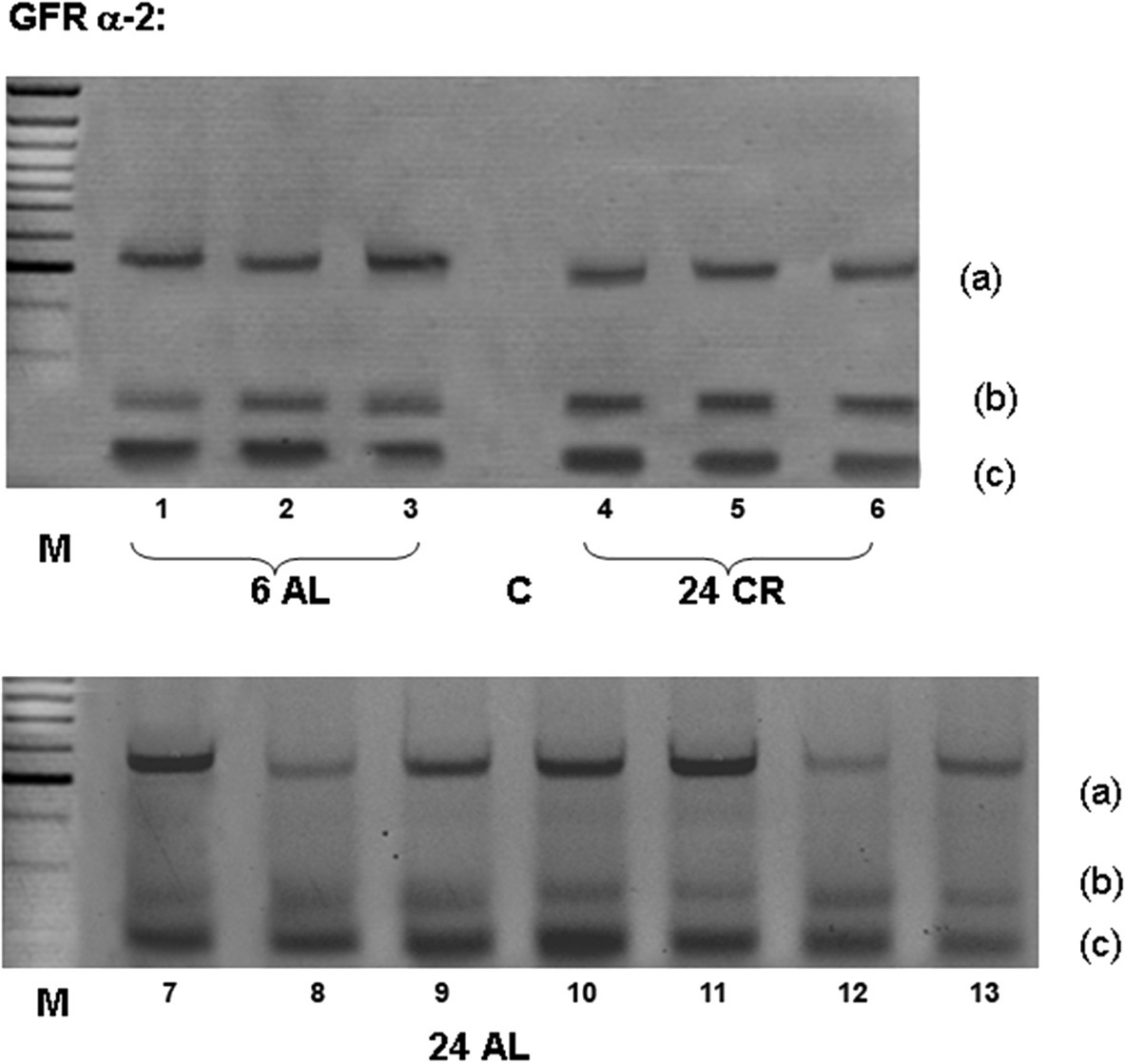
Expression of mRNA encoding the three isoforms of GFRα-2 in the muscularis externa of 6 and 24 month rats.

**Figure 4:**
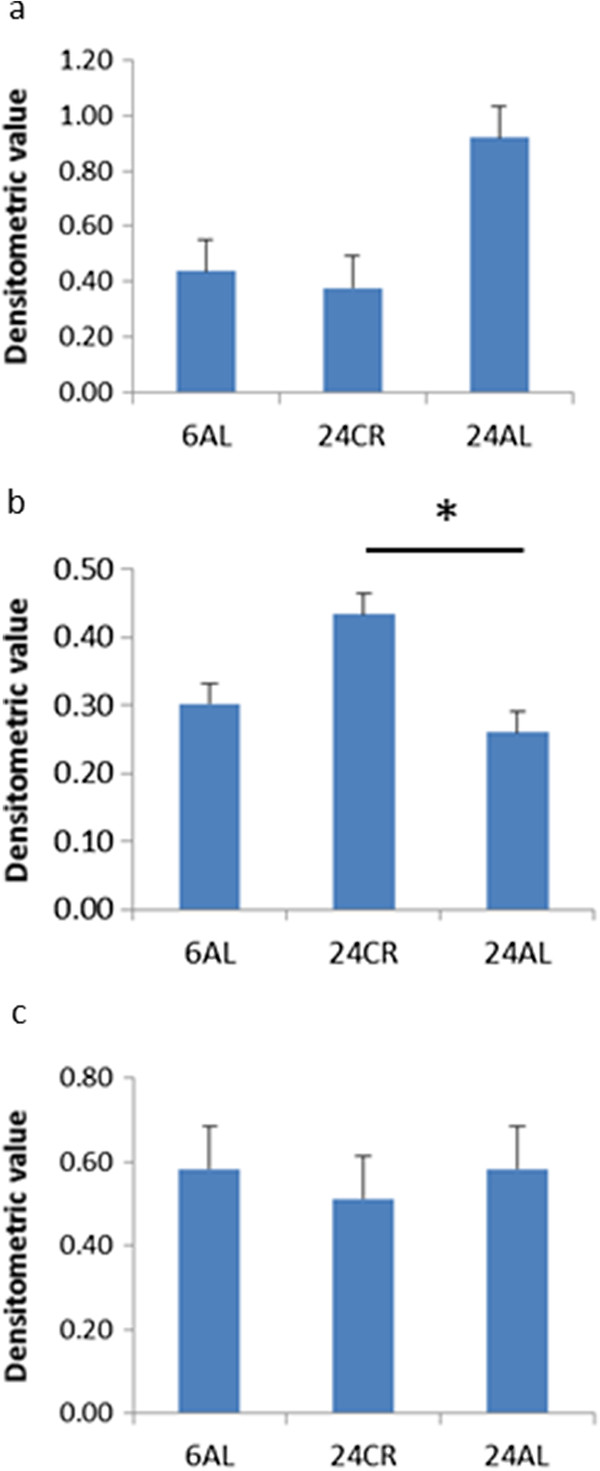
**Densitometric analysis of (a) GFR α-2(a) (b) GFR α-2(b) and (c) GFR α-2 (c) RT-PCR gels standardised against β-actin expression.** Levels are in arbitrary units, bars show standard errors. * p ≤ 0.05.

The GDNF ligand-binding receptor, GFR α-1, was also found to be expressed in all groups of animals studied (Figure 1), but levels were elevated in samples from 24 month AL-fed animals (Figure 2d). GFR α-2, the specific ligand-binding receptor for NTN was also found to be expressed in adult and ageing muscularis externa, with the GFR α-2(b) isoform being expressed at lower levels than the other two isoforms (Figures 3 and 4). Densitometric measurements showed a change in the relative abundance of different isoforms of the receptor in ageing AL fed animals (Figure 4). In 24 month AL-fed animals there was a non-significant increase in the level of the GFR α-2 (a) isoform; and a significant decrease in the level of the GFR a-2 (b) isoform (Figure 4). The GFR α-2(c) isoform, however, was found to be expressed at a similar level in all samples (Figure 4).

**Figure 5:**
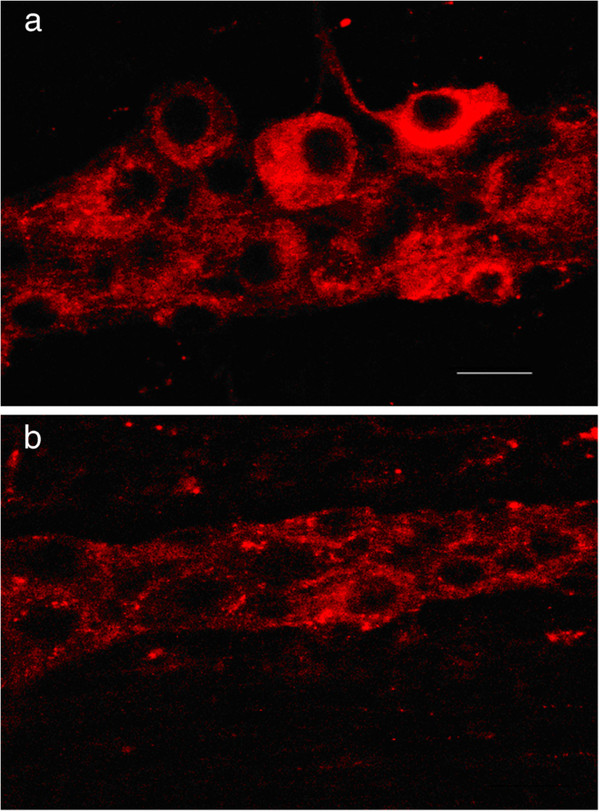
**Immunofluorescence micrographs showing (a) c-Ret and (b) GFR α-1 immunoreactivity in myenteric neurons from 1.5 year AL-fed rat ileum.** Scale bar = 25 μm.

In order to determine if c-Ret and GFR α − 1 receptor proteins were also expressed in the ageing ENS, whole mount preparations of the myenteric plexus from 18 month AL-fed animals were immunolabelled with antisera raised against c-Ret or GFR α-1 (antisera against GFR α-2 were not available at the time these samples were processed). Both receptors were found to be expressed widely by neurons within the myenteric plexus (Figures 5a and b).

These results indicate that both GDNF and NTN continue to be expressed in the muscularis externa of the rat ileum during ageing, and that there are some differences in expression between AL and CR animals; GDNF levels being elevated in the muscularis externa of AL-fed animals. Ageing myenteric neurons also continue to express the receptor proteins needed for GDNF actions; increased levels of GFR α-1 and decreased levels of GFR α-2 beng measured in ageing AL-fed animals. These changes in GDNF and GFR α − 1 could indicate compensatory changes in the cells of the muscularis externa due to increased stress of neurons in AL-fed animals [8]. A decrease in the levels of GFR α-2 could indicate reduced efficacy of NTN binding and subsequent signalling in 24 month AL-fed animals, so could contribute to neuronal losses reported in these animals [8]. It is important to note that there was some variability in receptor expression between samples. Future work, such as analysis of expression by single neurons isolated by laser-capture microdissection, would provide valuable information about whether the responsiveness of individual neurons could vary as a result of variation in receptor expression.

### Effects of NT-3 and GDNF on antioxidant enzyme expression by enteric ganglion cells in dissociated culture

**Figure 6:**
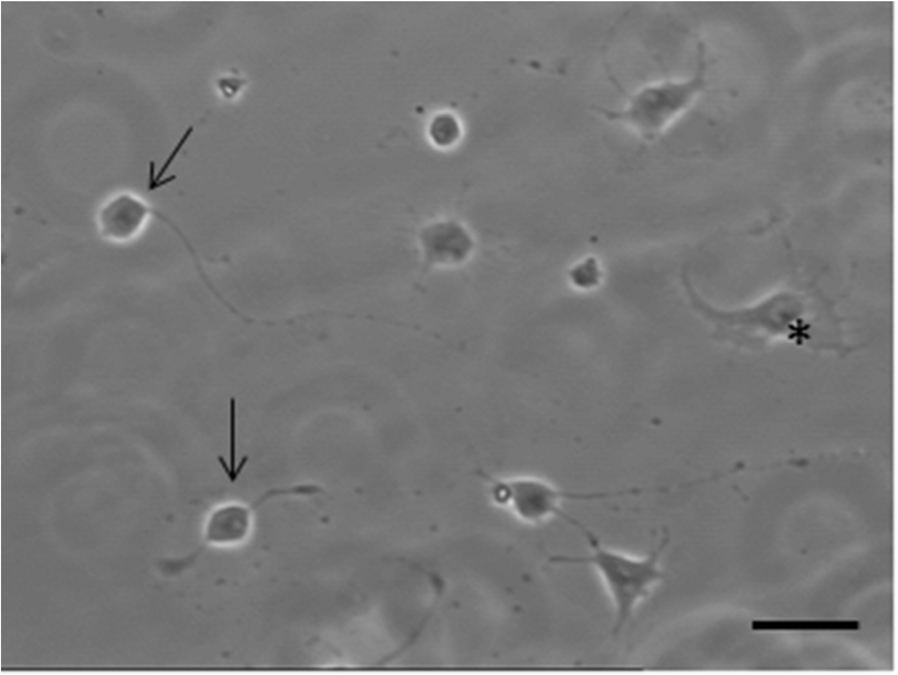
**Phase contrast image of dissociated culture after 24 hours*in vitro*without either neurotrophic factors or H**_**2**_**O**_**2**_*.*Neuronal cell bodies, extending neurites (arrows) and glial cells (*) can be seen. Scale bar = 25 μm.

**Figure 7:**
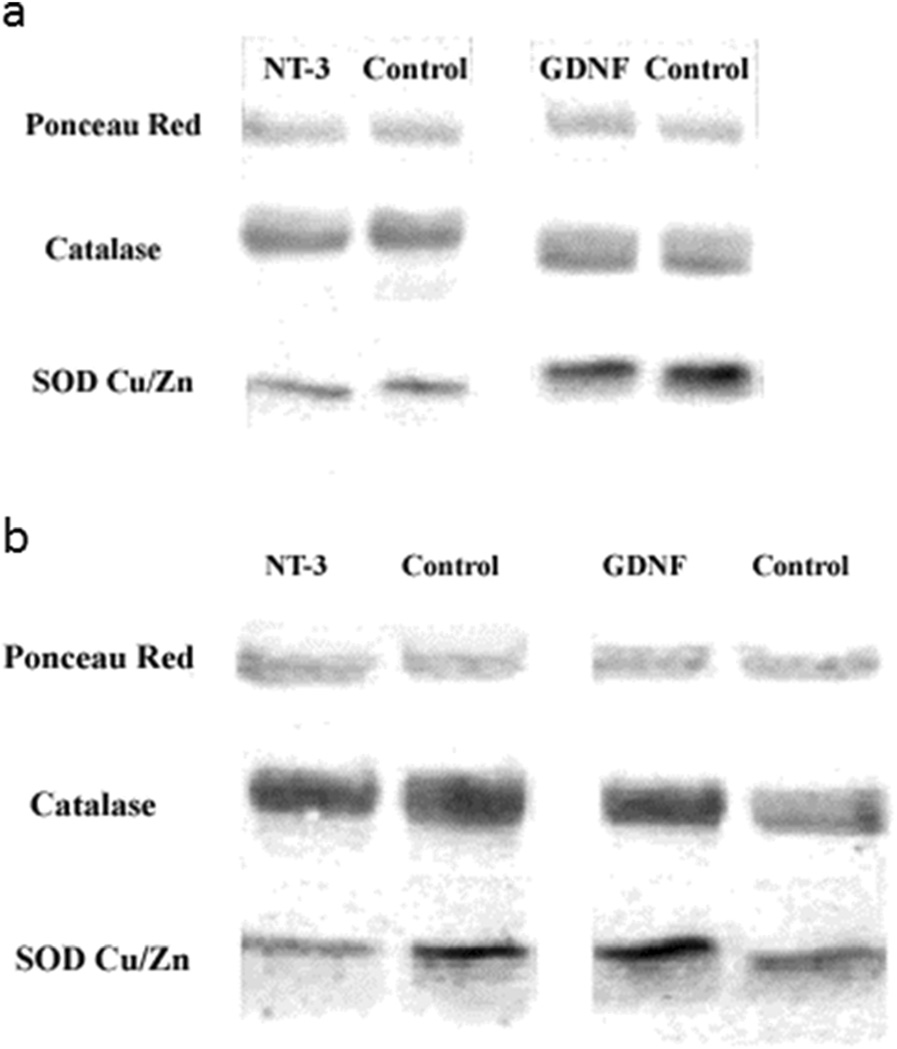
**Examples of Western blots of lysates from sister cultures grown in the presence of NT-3 or GDNF for 12 hours (a) or 36 hours (b) prior to protein extraction, using antibodies directed against catalase (middle row)or superoxide dismutase Cu/Zn (bottom row). 5 μg of protein extracts was loaded on to each gel.** To ensure equal protein content on each lane after electro blotting membranes were incubated with Ponceau red (top row), and protein amounts were estimated using densitometry.

**Figure 8:**
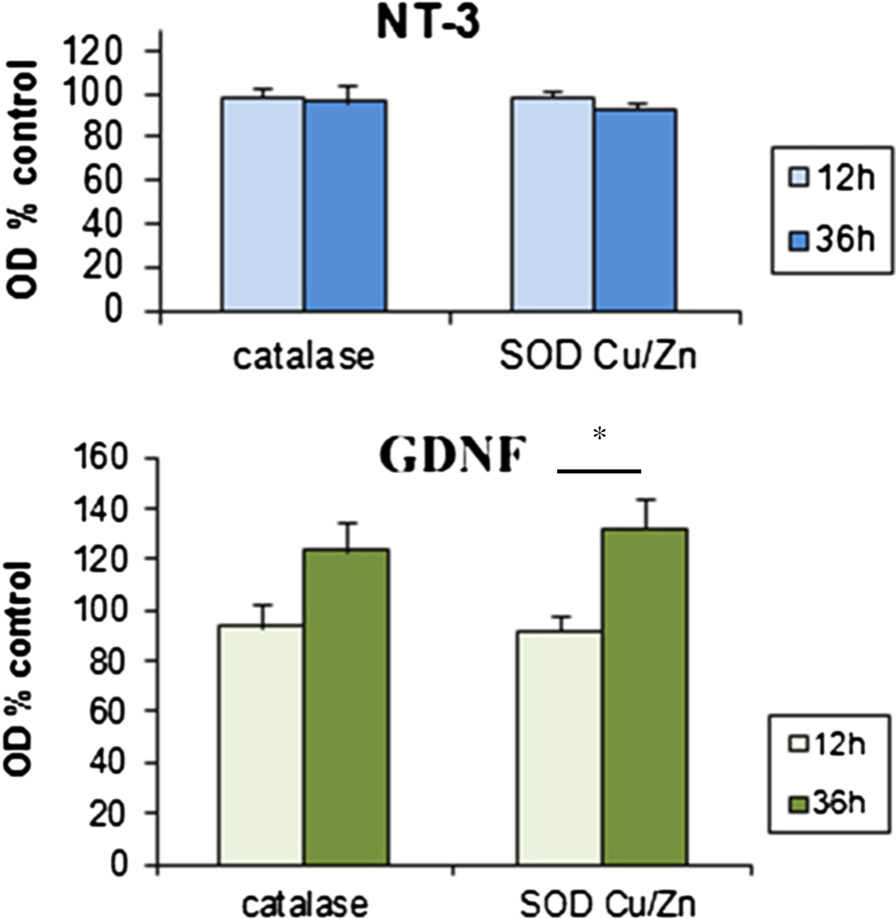
**Densitometric analysis of western blot membranes loaded with equal amounts of protein and expressed as a percentage of respective controls.** Effects of (a) NT-3 and (b) treatment GDNF after 12 and 36 hours are shown. OD readings were standardized against the values obtained for total protein staining of respective membranes. Data presented are means (± SEM) from at least 5 separate experiments. * p < 0.05.

Possible changes in the levels of catalase and Cu/Zn SOD were examined in dissociated cell cultures obtained from myenteric ganglia that had been separated from the surrounding smooth muscle by established methods [9], and see Additional file 1. These cultures contain both enteric neurons and glial cells (Figure 6). Responses to neurotrophic factor treatment were assessed by Western blotting after 12 and 36 hours exposure to the factors. Samples from NT-3 treated cultures showed no changes in the levels of catalase or Cu/Zn SOD after 12 hours or 36 hours incubation with the factor compared to untreated controls (Figure 7a and b). In contrast, cultures grown with GDNF showed an increase in the levels of both enzymes, especially that of Cu/Zn SOD (Figure 7a and b). These results were confirmed by subsequent densitometric analysis of the membranes (Figures 8a and b). The increase in enzyme levels in the GDNF-treated cultures was not due to a change in the relative proportions of neurons and glial cells in the cultures, as demonstrated by cell counts in cultures immunolabelled with the neuronal marker PGP9.5. The ratios of neurons to glial cells in the cultures grown under different conditions showed no significant change and are shown in Table 1. 

**Table 1:** Effect of factor treatment on the mean number of neurons, and the neuron to glial cell ratio in cultures of enteric ganglion cells after 12 and 36 hours incubation

	**Control**		**NT-3**		**GDNF**	
	**Neurons**	**Neurons/ glia**	**Neurons**	**Neurons/ glia**	**Neurons**	**Neurons/ glia**
12 hours	159 (±0.36)	0.31	169 (±0.41)	0.36	176 (±0.55)	0.37
36 hours	119 (±0.32)	0.23	151 (±0.37)	0.29	195 (±0.44)	0.34

Cultures established as described in the Methods were grown in the presence of NT-3 or GDNF, both at 10 ng/ml concentration, fixed after 12 or 36 hours and subsequently stained with PGP 9.5. Values represent the mean of neuronal numbers counted in 3 separate experiments, each treatment consisting of 3 replicas per experiment. Values in parentheses represent ± SEM.

These results indicate that GDNF, but not NT-3 can promote increased expression of the antioxidant enzymes catalase and Cu/Zn SOD by enteric ganglion cells in this dissociated cell culture model. This finding is in keeping with previous reports that some neurotrophic factors increase the levels of antioxidant enzymes in other cell types, including other neuronal populations e.g. [11]. Future work, to analyse effects of neurotrophic factors on the levels of Mn SOD and other antioxidants must also be performed. The lack of an effect of NT-3 in this system was somewhat unexpected, since it has been shown to promote survival of both ageing myenteric neurons in isolated plexus samples [8] and young enteric ganglion cells in dissociated cell culture [9], when these preparations were exposed to menadione- and hydrogen-peroxide, respectively.

The results described here demonstrate that neurotrophic factors continue to be expressed in the ageing gut, and can enhance the expression of antioxidant enzymes by enteric ganglion cells. The effect of GDNF on antioxidant enzyme levels observed here is in part in keeping with previous evidence showing that GDNF reduced ROS generation by ageing myenteric neurons in isolated myenteric plexus preparations from CR animals [8], and also that this neurotrophic factor prevented meniadione-induced neuronal apoptosis in this system [8]. However, in previous work, GDNF was not found to have protective effects on menadione-treated samples from ageing AL-fed animals [8]. A possible explanation of a lack of a protective effect of GDNF on neurons from AL-fed animals despite maintained receptor expression is that downstream signalling or other pathways may be disrupted or impaired in neurons from ageing AL-fed animals. Thus the increased expression of GDNF and GFR-a-1 observed in 24 month AL fed animals seen in the present study could be a compensatory mechanism to overcome downstream changes.

These results therefore support the hypothesis that disruption of neurotrophic factor signalling may play an important role in the ageing of the ENS, and indicate several possible lines of future study. In this context it will be important to investigate if changes in expression of neurotrophic factor receptors or activation of downstream signalling pathways occur at the level of individual neurons in the ageing ENS. The pathways known to be involved in the protective effects of GDNF in the ENS include the PI3K/Akt pathway; for example activation of the PI3K/Akt pathway by GDNF is involved in the rescue of enteric neurons from hyperglycemia-induced neuropathy [12]. Design of agents that may promote neurotrophic factor expression, or allow targeted delivery of factors or mimetics may therefore be valuable in preventing or ameliorating enteric neuropathy [13-17].

## Methods

See Additional file 1.

## Abbreviations

GDNF : Glial cell line derived neurotrophic factor; GFR : Glial cell line derived neurotrophic factor receptor; NT-3 : Neurotrophin-3; NTN : Neurturin.

## Competing interests

The authors declare that they have no competing interests.

## Authors’ contributions

N.F.D. performed the analysis of neurotrophic factor expression and contributed to drafting of the manuscript. K.K. performed the analysis of antioxidant enzymes in culture preparations, densitometry and contributed to drafting of the manuscript. A.T.S. contributed to analysis of neurotropic factor expression and culture experiments and to drafting of the manuscript. M.J.S. instigated the study, participated in its design and drafted the manuscript. All authors read an approved the final manuscript.

## Supplementary Material

Additional file 1 Saffrey et al Revised 9 7 12 Methods. Click here for file
