# Supplementary material for: Ageing of enteric neurons: oxidative stress, neurotrophic factors and antioxidant enzymes
Source: Chem Cent J. 2012 Aug 2;6:80. doi: 10.1186/1752-153X-6-80 (PMC3469348; doi:10.1186/1752-153X-6-80)
Supplement: Additional file 1 — Saffrey et al Revised 9 7 12 Methods. [file 1752-153X-6-80-S1.docx]

**Korsak, Dolashad and Silva and Saffrey (Saffrey et al. revised)**

**Methods**

*Animals* Tissue samples for RNA analysis were obtained from male Sprague Dawley rats from a colony maintained at the Royal Free and University College Medical School. Animals were reared up to 6 months of age with *ad libitum* (AL) access to BEEKAY Rat and Mouse standard 1 diet (B&K Universal Ltd, Hull, UK), composed of 19% crude protein, 5% oil, 4% fibre and 72% dry matter. The caloric content was 16.16 mJ/kg and the rats consumed an average of 50-60g per day, i.e. 0.80-0.96mJ per day. From 6 months of age, some animals were transferred over a period of 4 weeks to a calorically- restricted (CR) diet of 25g per day (0.40mJ), while others remained on the AL dietary regime. Dietary restriction and all other conditions were undertaken in compliance with Home Office legislation under the Animals (Scientific Procedures) Act, 1986.

*C-Ret and GFR α-1 immunolabelling:* Following dissection, the samples were placed in cold phosphate buffer (PBS pH 7.3).Tissues were cut open along the mesenteric border and pinned onto Sylgard® resin. All preparations were washed for 2 minutes with an acetic acid rinse (distilled water containing 5% acetic acid and 0.9% sodium chloride). Two fixatives were used throughout the experiments: 4% paraformaldehyde (PFA) in dPBS (diethlypyrocarbonate-treated PBS) and Zamboni’s (dPBS containing 4% PFA and 15% saturated picric acid). Different fixation periods and temperatures were tested and subsequently the samples were fixed at room temperature for 2 hours with either of the fixatives. Tissue fixed in Zamboni’s was then washed in 80% ethanol followed by dPBS and the 4% PFA-fixed tissues were only washed with dPBS. All washing steps were carried out for 10 minutes at room temperature and repeated three times. Tissues were then permeabilised using dPBS containing 0.5% Triton X100 for 1 hour at room temperature.

The muscularis externa of the ileum was then peeled away from the submucosa and mucosa under a dissecting microscope. The circular muscle was then removed by microdissection, leaving the longitudinal muscle and the attached myenteric plexus for immunohistochemical studies.

For immunolabelling, non-specific binding sites were blocked by incubation in 2% goat serum in PBS for 1 hour at room temperature. Tissues were then incubated overnight at 4^o^C in primary antibodies raised in goat (polyclonal anti-c-Ret, R&D Systems, 1:250; polyclonal anti-GFR α1, R& D Systems, 1:250), washed in PBS, and then incubated in Rhodamine Red^TM^X-conjugated donkey anti-goat IgG (Jackson, USA, 1:400) for 1 hour at room temperature. After further washes, tissues were mounted using either Citifluor anti-fade mountant (City University, UK) or Mowiol^TM^, semipermanent mounting medium and examined under X40, X20 and X10 objectives using either a Zeiss Axiophot light microscope fitted with a standard FITC filter set or observed under Leica confocal microscope.

## *Total RNA extraction and RT-PCR:*

Tissues were rapidly collected into sterile cryovials and flash frozen for RNA extraction. Total RNA was extracted from the peeled muscularis externa of individual animals of 6 and 24 months of age using the single-step guanidinium thiocyanate and acidic phenol method. The RNA samples were then reverse transcribed. First strand synthesis was performed using random hexamers (Amersham Life Sciences). 42 μg of the total RNA extracts were incubated for 1 hour at 42 ^0^C in 42 μl of total reverse transcription (RT) reaction mixture including DNase-1 (Gibco BRL), RNase inhibitor (Promega) and 2.5 U/μl SuperScript II RT^TM^ enzyme (Gibco BRL). For PCR, 1 μl of cDNA was mixed with gene specific primers (see Table 1) and 2.5 U thermostable Taq polymerase (Promega) in a total reaction volume of 25μl. PCR reactions were carried out in a PT-100 thermal cycler (MJ Research Inc. UK), with a cycling parameter of 96 ^0^C (5 min), 80 ^0^C (1 min), and 37 cycles of; 94 ^0^C, 58 ^0^C and 72 ^0^C at 40 seconds each followed by the final elongation step of 72 ^0^C (5 min). β-actin was used for external standardisation of the samples and all PCR reactions were initiated with a hot start. PCR products were analysed by gel electrophoresis; band density was measured using Image software, and subsequently analysed by two-way analysis of variance.

*Primer design:*

The nucleotide sequence of each rat gene of interest was initially retrieved from the database using the National Center for Biotechnology Information [1]. The sequences were then used in the design of primers for PCR. The primers used are shown in Table 1. The β-actin primers were purchased from Promega; other primers were synthesised by MWG Biotech.

# *Cell culture:*

Enteric ganglion cell cultures were prepared as previously described [2,3]. Briefly, small intestines of 7 day old Sprague-Dawley rats were harvested and placed in Hanks balanced salt solution (HBSS, Sigma) containing gentamycin and metronidazole. After removing the mesentery, the *muscularis externa* with embedded enteric ganglion cells was separated by peeling from the rest of the gut and incubated for 30 minutes in collagenase type II (1mg/ml in HBSS containing 10 μg/ml DNase) at 37°C. Subsequently the tissue was vortexed and incubated with fresh enzyme for another 30 minutes. Freed pieces of plexus were collected under a dissecting microscope, placed in trypsin-EDTA solution (Sigma), incubated for 15 minutes and dispersed by passage through a 25 gauge needle to obtain a single cell suspension. Cells were seeded in 150 μl of 199 medium with N1 supplements (N 199) containing 10% fetal calf serum (Sigma) at 2 X 10^4^ cells per 13mm diameter glass coverslip coated with poly L lysine. After 1 hour incubation at 37°C, 2.5 % CO_2_ a further 850 μl of 199 N1 medium was added. 16 hours later medium was removed and replaced with serum free 199 N1. Cells were supplemented with desired concentrations of factors 24 hours after plating.

*Proteins extraction and Western blot analysis:*

Protein lysates were prepared by washed cultures three times with cold PBS and scraping into NP40 lysis buffer (PBS, 1%NP40 detergent, NaCl, Tris pH 8.0, protease inhibitor cocktail (Boeringer). Subsequently samples were incubated on ice for 30 minutes and spun at 10000 rpm, 4°C for 10 minutes and supernatants were collected and stored at -20°C for protein quantification. Protein assay was performed using Bio Rad protein assay kits, according to manufacturer’s protocol. Standard curves were prepared for each session of protein determination from known quantities of bovine serum albumin (Sigma).

Proteins were separated on 15% SDS-PAGE gels in electrode buffer (25mM Tris, 192mM glycine, 0.1% SDS) at 120V for 3 hours and transferred to nitrocellulose membranes by electroblotting in transfer buffer (25mM Tris, 192mM glycine, 0.1% SDS, 20% methanol) at 100V for one hour. Equal protein loading was checked by incubating membranes containing transferred proteins with Ponceau red (Sigma).To obtain an estimate of the amount of catalase and SOD Cu/Zn detected on the membranes, and also to check the sensitivity of assay, standard curves were prepared using from 50 to 800ng of purified catalase or SOD Cu/Zn protein (Sigma). Membranes were blocked in PLT (PBS, 5% non fat dry milk, 0.1% Tween 20) overnight at 4°C, and processed for immunoblotting. Briefly, blots were incubated with primary antibodies (Anti Catalase and anti SOD Cu\Zn Calbiochem, each at 1:2000), diluted in PLT for two hours at room temperature, washed three times, 10 minutes each time with PLT and incubated with secondary antibodies conjugated with horseradish peroxidase for one hour at room temperature with two subsequent washes in PBS containing 1% Tween 20 and a final 10 minutes wash with PBS. Blots were developed using chemiluminescence kit (Amersham). Exposed films were processed on an automated Kodak X-OMAT processor and digitised using alpha imager software; optical density was measured using Scion Image software (Scion Corporation).

*Densitometry* was used in order examine the differences between bands obtained in RCR gels and Western blots. Briefly, a diagonal strip across the images containing the bands was selected. Subsequently, software package measured intensity of bands and prepared a densitometric graph with peaks which area depends on the intensity of bands. Optical density (OD) values, corresponding to the areas under peaks were then gathered from several western blot membranes and used to calculate mean OD value of a band obtained for a given protein.

*Statistical analysis:* Data were analysed by one-way analysis of variance (ANOVA), at p < 0.05 using Origin software package.

**Table 1**: **Primer sequences used**

| **Gene:**  (Genbank accession number) | **Forward primer:** | **Reverse primer:** |
| --- | --- | --- |
| GDNF  (L15305) | GTCTACGGAGACCGGATCCGAGGTG | GAGTGGTCTTCGGCGGGC |
| NTN  (AF184922) | AGGGTCTGCTCTTGGGTCACC | AAGGACACCTCGTCCTCATAGGC |
| GFR α-1  (U59486) | AGAGCTGCAGCACCAAGTACCGCAC | CGACCTGTACTTCTTACAGGTGTCG |
| GFR α-2  (AF005226) | GCCTTCTGCCTCTTCTTCTTTTTAG | ACGGTTGCAGATGGAGATATAAGAG |
| c-Ret  (10638065) | GCACACCTCTGCTCTATGTCC | AGTGTCATTGGCCAGGCTGT |
| β-actin | TCATGAAGTGTGACGTTGACATCCGT | CCTAGAAGCATTTGCGGTGCACGATG |

**References**

[1] NCBI: <http://www.ncbi.nlm.nih.gov>

[2] Saffrey MJ, Wardhaugh T, Walker T, Daisley J, Silva AT, **Trophic actions of neurotrophin-3 on postnatal rat myenteric neurons *in vitro***, Neurosci. Letters, 2000, **278**:133-136

[3] Korsak, K, Silva A.T., Saffrey, M.J. **Differing effects of NT-3 and GDNF on dissociated enteric ganglion cells exposed to hydrogen peroxide in vitro.** Neurosci. Letters 2012, **517:** 102– 106
